# Epidermal growth factor signaling through transient receptor potential melastatin 7 cation channel regulates vascular smooth muscle cell function

**DOI:** 10.1042/CS20200827

**Published:** 2020-08-04

**Authors:** Zhi-Guo Zou, Francisco J. Rios, Karla B. Neves, Rheure Alves-Lopes, Jiayue Ling, George S. Baillie, Xing Gao, William Fuller, Livia L. Camargo, Thomas Gudermann, Vladimir Chubanov, Augusto C. Montezano, Rhian M. Touyz

**Affiliations:** 1 Institute of Cardiovascular and Medical Sciences, University of Glasgow, Glasgow, U.K.; 2 Walther Straub Institute of Pharmacology and Toxicology, Ludwig-Maximilians Universität München, Goethestrasse 33, Munich 80336, Germany

**Keywords:** EGF, TRPM7, vascular smooth muscle

## Abstract

**Objective:** Transient receptor potential (TRP) melastatin 7 (TRPM7) cation channel, a dual-function ion channel/protein kinase, regulates vascular smooth muscle cell (VSMC) Mg^2+^ homeostasis and mitogenic signaling. Mechanisms regulating vascular growth effects of TRPM7 are unclear, but epidermal growth factor (EGF) may be important because it is a magnesiotropic hormone involved in cellular Mg^2+^ regulation and VSMC proliferation. Here we sought to determine whether TRPM7 is a downstream target of EGF in VSMCs and if EGF receptor (EGFR) through TRPM7 influences VSMC function.

**Approach and results:** Studies were performed in primary culture VSMCs from rats and humans and vascular tissue from mice deficient in TRPM7 (TRPM7^+/Δkinase^ and TRPM7^R/R^). EGF increased expression and phosphorylation of TRPM7 and stimulated Mg^2+^ influx in VSMCs, responses that were attenuated by gefitinib (EGFR inhibitor) and NS8593 (TRPM7 inhibitor). Co-immunoprecipitation (IP) studies, proximity ligation assay (PLA) and live-cell imaging demonstrated interaction of EGFR and TRPM7, which was enhanced by EGF. PP2 (c-Src inhibitor) decreased EGF-induced TRPM7 activation and prevented EGFR–TRPM7 association. EGF-stimulated migration and proliferation of VSMCs were inhibited by gefitinib, PP2, NS8593 and PD98059 (ERK1/2 inhibitor). Phosphorylation of EGFR and ERK1/2 was reduced in VSMCs from TRPM7^+/Δkinase^ mice, which exhibited reduced aortic wall thickness and decreased expression of PCNA and Notch 3, findings recapitulated in TRPM7^R/R^ mice.

**Conclusions:** We show that EGFR directly interacts with TRPM7 through c-Src-dependent processes. Functionally these phenomena regulate [Mg^2+^]_i_ homeostasis, ERK1/2 signaling and VSMC function. Our findings define a novel signaling cascade linking EGF/EGFR and TRPM7, important in vascular homeostasis.

## Introduction

Transient receptor potential (TRP) melastatin 7 (TRPM7) is a ubiquitously expressed bi-functional protein comprising a cation channel and a C-terminal α-kinase domain [1,2]. The ion channel is highly permeable to divalent cations, including Mg^2+^, Zn^2+^ and Ca^2+^ [3]. The kinase domain of TRPM7 phosphorylates several targets including calpain-II, annexin-1, myosin IIA, phospholipase Cγ2 (PLCγ2), eukaryotic elongation factor 2-kinase (eEF2-k) and Smad2 [1,4]. Mg^2+^ is an essential divalent cation that is involved in many cellular processes such as DNA stability, cell metabolism and cell growth [4,5]. Exact mechanisms underlying intracellular Mg^2+^ homeostasis remain unclear, but several channels and transporters have been implicated including TRPM6, TRPM7 and SLC41A1 [6]. Of these, TRPM7 is most widely expressed and is particularly important in the cardiovascular system [7]. TRPM7 is essential in the prenatal development because a null mutation in the TRPM7 locus triggers early embryonic mortality [8]. Early cardiac TRPM7-deletion results in congestive heart failure and death by hypoproliferation of myocardium [9]. TRPM7 is required to maintain cardiac automaticity in sinoatrial node (SAN) and regulates SAN fibrosis induced by angiotensin II (Ang II) through Smad signaling [9,10]. Using mice heterozygous for the truncation mutation in TRPM7 (TRPM7^+/Δkinase^), we demonstrated that TRPM7 deficiency is associated with cardiovascular inflammation and fibrosis and increased susceptibility to Ang II-induced endothelial dysfunction and hypertension [11,12]. At the cellular level, we showed that in vascular smooth muscle cells (VSMCs) TRPM7 attenuates aldosterone-induced pro-inflammatory signaling [13]. We also found that TRPM7 plays an essential role in VSMC proliferation in a Mg^2+^-dependent fashion [14] and control of vasoactive agents such as Ang II and growth factors, including epidermal growth factor (EGF) [15].

EGF binds to its EGF receptor (EGFR) tyrosine kinase and is a key regulator of vascular function [16]. Activation of EGFR is associated with autophosphorylation at key tyrosine residues, leading to activation of multiple downstream signaling cascades such as the Src family kinases and mitogen activated protein (MAP) kinases [17]. In addition, in VSMCs, EGF/EGFR activates Ca^2+^ channels such as TRP classical (TRPC) type 3 and 6 and TRPM4 channels, and voltage-dependent calcium channels, thereby potentiating Ca^2+^-regulated myogenic tone [18,19]. EGF has also been shown to influence TRPM6 in human embryonic kidney (HEK) 293 cells (HEK-293) cells, a human embryonic kidney cell line and TRPM7 in A549 cells, a human alveolar adenocarcinoma cell line [20,21]. Activation of EGFR promotes VSMC proliferation, migration, inflammation and fibrosis, processes involved in vascular remodeling in cardiovascular diseases such as hypertension and atherosclerosis [16]. Through its mitogenic signaling and dysregulated trafficking, EGFR has also been defined as an oncogenic driver that plays an important role in the progression of many epithelial cell cancers [22].

Inhibition of EGFR and its downstream signaling targets ameliorate endothelial dysfunction and vascular remodeling in experimental models of hypertension [19]. In addition, molecular targeted inhibitors of EGFR are currently used as anticancer therapies [22,23]. Small molecule tyrosine kinase inhibitors, such as gefitinib, afatinib, erlotinib and osimertinib and anti-EGFR monoclonal antibodies including cetuximab and panitumumab, prolong survival in cancer patients [24]. An unexpected adverse effect of EGFR inhibition in cancer patients is hypomagnesemia [25,26]. The severity of hypomagnesemia is associated with efficacy of EGFR inhibition and hypomagnesmia has been suggested to be a biological marker of therapeutic effectiveness in some cancers [27,28]. The exact cause of EGFR inhibitor-induced hypomognesemia remains unclear. *In vitro* studies in HEK-293 cells suggest that abnormal TRPM6-induced Mg^2+^ may be important, while *in vivo* studies failed to show a role for renal TRPM6 in plasma levels of Mg^2+^ [20,29,30].

Given the important role of EGF, Ca^2+^, Mg^2+^ and TRPM7 in VSMCs, we sought to evaluate whether EGF signals through TRPM7-dependent processes and if this influences VSMC function and vascular morphogenesis. We also investigated molecular mechanisms linking EGFR and TRPM7 in VSMCs, focusing on c-Src as a putative point of cross-talk.

## Materials and methods

### Animals

All experimental procedures on rats and mice that were performed at the University of Glasgow were approved by the University of Glasgow Animal Welfare and Ethics Review Board in accordance with the United Kingdom Animals Scientific Procedures Act 1986 (Licence No. 70/9021) and the ARRIVE Guidelines. Tissues from TRPM7 ‘kinase-dead’ mice (TRPM7^R/R^) were provided by Dr. Chubanov, Ludwig-Maximilians Universität München. TRPM7^R/R^ mice were handled according to the European Union Animal Welfare Act and the local councils on animal care (permits AZ: 55.1-8791-14.718 and 55.2-1-54-2532-180-2016 from Government of Oberbayern).

Studies were performed in primary culture VSMC from Wistar–Kyoto (WKY) rats, TRPM7-kinase deficient mice (TRPM7^+/Δkinase^) and humans. Some experiments were also conducted in intact aorta from TRPM7^+/Δkinase^ mice [8,12] and from mice with global kinase-dead point mutation in TRPM7 (TRPM7^R/R^) [3]. These mice have been comprehensively phenotyped [11].

### WKY rats

WKY rats were housed in individual cages under controlled conditions of 22–24°C and a 12-h light/dark cycle with freely accessible food and tap water. Sixteen-week-old male WKY rats were killed and mesenteric arteries were isolated for primary culture of VSMCs.

### Wildtype and TRPM7 kinase-deficient mice

Wildtype (WT) mixed background (C57BL/6J and SV129) TRPM7 kinase-deficient mice (TRPM7^+/Δkinase^) mice were generated as previously described [8,11]. In TRPM7^+/Δkinase^ mice, TRPM7 protein is truncated immediately upstream of the α-kinase domain associated with reduced channel activity [8]. 18-22 week-old male mice were killed aortae collected, and mesenteric arteries dissected for isolation of primary culture of VSMCs. Aortic segments were snap-frozen for later analysis.

### WT C57BL/6 and TRPM7 kinase-dead mice

Aortic tissue from WT C57BL/6 and TRPM7 kinase-dead mice (TRPM7^R/R^) mice has been reported previously [31,32]. The TRPM7^R/R^ locus contains a ‘kinase-dead’ K1646R point mutation, which specifically abrogates the catalytic activity of TRPM7 kinase domain in the whole organism.

### Cell culture

#### VSMCs from WKY rats

Primary VSMCs were isolated from mesenteric arteries of four to eight animals by enzymatic digestion as we previously described [33]. Rat VSMCs (rVSMCs) were cultured in Dulbecco’s Modified Eagle’s Medium (DMEM) supplemented with 10% fetal bovine serum (FBS) and Penicillin/Streptomycin (50 µg/ml), all from Life Technologies. Confluent cells were rendered quiescent overnight in DMEM containing 0.5% FBS before experiments. rVSMCs were studied at passages 4–8.

#### VSMCs from humans

Studies were conducted under ethics approval obtained from the West of Scotland Research Ethics Service (WS/12/0294) and all subjects provided written informed consent. Small arteries were dissected from surplus surgical tissue of patients receiving elective craniofacial surgeries at the Craniofacial/Oral and Maxillofacial Unit, Queen Elizabeth University Hospital, Glasgow.

Primary human VSMCs (hVSMCs) were isolated from 12 individuals as we previously described [34] and were cultured in DMEM supplemented with Smooth Muscle Growth Supplement (SMGS) (Life Technologies, Paisley, U.K.) and antibiotics. Confluent cells were rendered quiescent overnight using DMEM containing 0.5% FBS before experiments. hVSMCs were studied up to passages 5–8.

#### VSMCs from WT and TRPM7^+/Δkinase^ mice

Mesenteric beds were dissected from four to eight animals from each group and primary VSMCs were isolated by enzymatic digestion [34]. Mouse VSMCs (mVSMCs) were cultured in DMEM containing 10% FBS and antibiotics. Confluent cells were rendered quiescent overnight with DMEM containing 0.5% FBS before experiments. mVSMCs were studied at passages 3–7.

#### Experimental protocols

Quiescent VSMCs were stimulated with EGF (50 ng/ml; R&D Systems) (5 min to 24 h). In some experiments, VSMCs were pre-exposed (for 30 min) to pharmacological modulators including gefitinib (EGFR inhibitor that reversibly binds to the tyrosine kinase domain of EGFR and inhibits receptor autophosphorylation by preventing its binding to ATP, 1 µM), apamin (small conductance calcium-activated potassium channel (SK channel) inhibitor, 1 µM), PP2 (c-Src inhibitor, 10 µM), PD98059 (MEK1/2 inhibitor, 20 µM), 2-Aminoethoxydiphenyl borate (2-APB) (non-specific TRPM7 inhibitor, 30 µM) and NS8593 (specific TRPM7 inhibitor, 40 µM) [35]. Concentrations used were based on previous studies [36,37]. Considering potential non-specificity of TRPM7 pharmacological inhibitors, we used two different agents: 2-APB and NS8593.

#### Immunoblotting

VSMCs and tissues were homogenized in lysis buffer containing Tris 50 mM, pH 8.0, NaCl 150 mM, Triton X-100 1%, SDS 0.1%, supplemented with phenylmethylsulfonyl fluoride (PMSF) 1 mM, pepstatin A 1 µg/ml, leupeptin 1 µg/ml, aprotinin 1 µg/ml (Sigma–Aldrich), sodium fluorate 10 mM (AnalaR Normapur; VWR International), and sodium orthovanadate 1 mM (Alfa Aesar). Total protein lysate was sonicated, followed by centrifugation at 12000 rpm for 15 min. Supernatants were collected and protein concentration was determined using the DC protein assay kit (Bio-Rad Laboratories). Proteins (20–30 µg) were separated by electrophoresis on polyacrylamide gels and transferred to nitrocellulose membranes. For proteins with MW higher than 150 kDa, such as Notch3 (270 kDa), TRPM7 (210 kDa) and TRPM6 (210 kDa), we used 7.5% SDS/PAGE gel electrophoresis. Proteins lower than 150 kDa such as ERK (42 kDa), p38 MAPK (38 kDa), Src (60 kDa) and PCNA (35 kDa), we used 10% SDS/PAGE gel electrophoresis. Non-specific bindings sites were blocked with 5% (w/v) non-fat dried milk solubilized in Tris-buffered saline (TBS) solution with 0.01% (v/v) Tween-20 (TBS-T) for 1 h at room temperature. Membranes were probed overnight at 4°C with the following primary antibodies diluted (1:1000) in TBS-T containing 3% (m/v) bovine serum albumin (BSA; Sigma–Aldrich,): TRPM7 (cat.#: ab245408, specific for humans and mice), TRPM6 (cat.#: ab79227), SLC41A1 (cat.#: ab83701) and α-tubulin (1:2000; cat.#: ab4074), phospho-EGFR (Y845; cat.#: ab194756), purchased from Abcam; TRPM7 (cat.#: MA5-27620, specific for humans, mice and rats), acquired from Invitrogen; EGFR (cat.#: 2646S), phospho-p38 MAPK (T180/Y182; cat.#: 9211S), p38 MAPK (cat.#: 9212S), phospho-ERK1/2 (T202/Y204; cat.#: 9101S), ERK1/2 (cat.#: 9102S), phospho-Src (Tyr^416^; cat.#: 2101S) and Src (cat.#: 2109), purchased from Cell Signaling Technologies; β-actin (1:10000; cat.#: A2228), purchased from Sigma–Aldrich. Phospho-TRPM7 (Ser1511, rabbit) antibody was produced by our collaborator Dr. Chubanov (Ludwig-Maximilians Universität München) and previously validated [38,39]. Subsequently, membranes were washed and then incubated with secondary fluorescence-coupled antibodies anti-mouse Alexa Fluor 680 or anti-rabbit Alexa Fluor 800 (Thermo Fisher) diluted (1:10000) in TBS-T containing 1% (m/v) BSA at room temperature for 1 h. An infrared laser scanner (Odyssey Clx, Li-COR) was used to visualize the membranes, and images were analyzed using the software Image Studio™ Lite free version (LICOR, Cambridge, U.K.).

#### Immunoprecipitation

VSMCs were harvested and homogenized in HEPES buffer (130 mM NaCl, 5 mM KCl, 1 mM CaCl_2_, 1 mM MgCl_2_, 10 mM d-glucose and 20 mM HEPES, pH 7.4), supplemented with PMSF 1 mM, pepstatin A 1 µg/ml, leupeptin 1 µg/ml, aprotinin 1 µg/ml (Sigma–Aldrich), sodium fluorate 10 mM (AnalaR Normapur; VWR International), and sodium orthovanadate 1 mM (Alfa Aesar). A total of 200 µl of VSMCs lysate containing 400 µg of protein was mixed with 2 µl of primary antibody (1:100) and the reaction mixture was rotated in a Stuart SB3 Rotator (Camlab Ltd) at a gentle speed overnight at 4°C. The following day, 15 µl of Protein A and 15 µl of Protein G (Santa Cruz Biotechnology) were added to the reaction mixture followed by rotation at 4°C for 4 h. The immunoprecipitation (IP) complex was then washed twice in lysis buffer followed by centrifugation at 14000 rpm for 30 s. The pellet was collected and prepared for immunoblotting.

#### Measurement of Ca^2+^ mobilization

Intracellular Ca^2+^ levels in VSMCs were assessed using the fluorescent Ca^2+^ indicator Cal-520 acetoxymethyl ester (Cal-520AM; Abcam, Cambridge, U.K.). Quiescent VSMCs cultured in 12-well plates were incubated with 10 μM Cal-520AM in DMEM containing 0.5% FBS at 37°C for 75 min, followed by 30 min at room temperature. Subsequently, the dye solution was replaced with HEPES buffer (130 mM NaCl, 5 mM KCl, 1 mM CaCl_2_, 1 mM MgCl_2_, 10 mM d-glucose and 20 mM HEPES, pH 7.4) for 30 min. In some experiments, inhibitors such as gefitinib (1 µM), NS8593 (40 µM) and 2-APB (30 µM) were added 30 min prior to stimulation with EGF (50 ng/ml). Fluorescent Cal-520AM-Ca^2+^ signals were recorded by an inverted epifluorescence microscope (Axio Observer Z1 Live-Cell imaging system, ZEISS, Cambridge, U.K.) at excitatory wavelength of 490 nm and emission of 535 nm. Data were acquired and analyzed using Zen Pro (ZEISS).

#### Measurement of intracellular Mg^2+^

Intracellular Mg^2+^ levels in VSMCs were measured using the specific fluorescent probe Magnesium Green AM (Thermo Fisher, cat.#:M3735) as we previously described [11]. Briefly, VSMCs were kept in Ca^2+^/Mg^2+^-free HEPES buffer (150 mM NaCl, 5 mM KCl, 10 mM d-glucose and 20 mM HEPES) and incubated with 5 μM Magnesium Green for 30 min at 37°C in dark and in constant gentle agitation to prevent cell adhesion. Subsequently, the stained cells were washed twice using phosphate-buffered saline (PBS) by centrifugation at 1200 rpm for 3 min at room temperature. The resulting cell pellet was resuspended in HEPES buffer with 1 mM Mg^2+^ (150 mM NaCl, 5 mM KCl, 10 mM d-glucose, 20 mM HEPES and 1 mM MgCl_2_). Cells were left at room temperature for 15 min to allow complete de-esterification of intracellular AM esters, followed by stimulation with EGF (50 ng/ml) for 5 min. In some studies, inhibitors such as gefitinib (1 µM), NS8593 (40 µM), apamin (1 µM), PP2 (10 µM) and 2-APB (30 µM) were added 30 min prior to EGF stimulation. Fluorescent signals of Magnesium Green were acquired using Flow Cytometry (FACS Canto II, BD Biosciences) and data were analyzed by FlowJo software (TreeStar, Ashland, U.S.A.).

#### Cell migration

VSMCs migration was assessed using the scratch-wound assay, a two-dimensional (2D) *in vitro* technique [40]. Confluent VSMCs cultured in 12-well plates were rendered quiescent overnight using DMEM with 0.5% FBS. The following day, a sterile pipette tip was used to scratch the cell monolayer, making a straight ‘wound’ with equal width. The medium was then replaced with fresh serum-free DMEM to remove floating cells and reduce the effects of cell proliferation. At zero-hour (0 h) time, three photos were taken from each well using a 10× objective (EVOS XL Core, Thermo Fisher). Cells were then incubated with EGF (50 ng/ml) in the presence and absence of inhibitors including gefitinib (1 µM), NS8593 (40 µM), 2-APB (30 µM) and PD98059 (20 µM). After 20 h, three photos were taken in the same areas for each well. Images were collected and analyzed using ImageJ software (National Institutes of Health, Bethesda, U.S.A.). Cell migration was calculated as the percentage of wound healing closure at 20 h relative to 0 h time point.

#### Proliferation assay

The cell tracking dye carboxyfluorescein succinimidyl ester (CFSE; cat.#: C34554, Thermo Fisher) was used in the proliferation assay. Following each cell division, the fluorescein-tagged cellular molecules are split evenly in two daughter cells, and cell proliferation can be assessed by measuring CFSE fluorescence. Briefly, isolated VSMCs were incubated with 5 µM CFSE in 1 ml of PBS containing 1% (v/v) FBS for 30 min at 37°C. After incubation, CFSE-labeled cells were washed twice using DMEM, and then plated at ∼30% confluence in six-well plates using DMEM with 10% FBS. The following day, medium was replaced with DMEM with 5% FBS and cells were then cultured for 72 h. During this period, cells were treated with EGF (50 ng/ml) in the presence and absence of inhibitors including gefitinib (1 µM), NS8593 (40 µM), 2-APB (30 µM) and PD98059 (20 µM). After 72 h, cells were harvested in PBS containing 1% FBS and CFSE fluorescence was detected with an FITC channel, using Flow Cytometry (FACS Canto II, BD Biosciences). Data were analyzed using the FlowJo software (TreeStar, Ashland, U.S.A.).

#### Histology

Aortic segments from WT and TRPM7^+/Δkinase^ mice were fixed in 10% buffered-formalin and embedded in paraffin. Sections (5-µm-thick) were stained with Hematoxylin and Eosin (HE). Briefly, aorta sections were deparaffinized and rehydrated in two 7-min changes of Histo-Clear (Fisher Scientific Ltd) and graded ethanol solutions (100, 95 and 70%, 7 min each), followed by 7 min in distilled water. Next, nuclei were stained with Harris’ modified Hematoxylin (Cell Pathology Ltd) for 2 min and then rinsed in running tap water for 5 min followed by 30 s in 70% ethanol. Cytoplasmic structures were stained using Eosin Y solution (Sigma–Aldrich) for 2 min. Consequently, tissue sections were rinsed in graded ethanol solution (95% for 30 s, 100% for 1 and 7 min separately), followed by two 5-min changes in Histo-Clear. Tissue sections were then mounted with glass coverslips using DPX non-aqueous mounting medium (Merck Millipore). Images were acquired using a light microscope (ZEISS) with 20× and 40× objective and were analyzed using ImageJ software.

#### Confocal microscopy

rVSMCs from WKY were seeded on cover glasses (13 mm, Thickness No. 0; VWR) at a density of 8 × 10^4^ cells per well in six-well plates. After stimulation, cells were fixed by 4% paraformaldehyde in PBS for 15 min and permeabilized with Triton X-100 (Sigma–Aldrich) in PBS for 4 min at room temperature. After three washes with TBS (150 mM NaCl, 20 mM Tris/HCl, pH 7.6), cells were blocked with 10% goat serum and 2% BSA (w/v) in TBS for 1 h at room temperature. Cells were then washed three times using TBS and incubated with specific primary antibodies to: TRPM7 (cat.#: MA5-27620; Invitrogen; 1:300) and EGFR (cat.#: 2646S; Cell Signaling; 1:300) overnight. The following day, cells were washed and then incubated with 1:400 diluted secondary antibodies Alexa Fluor 555 Goat anti-Rabbit IgG (cat.#: A-21428; Invitrogen) and Alexa Fluor 488 Goat anti-Mouse IgG (cat.#: A-11001; Invitrogen) for 2 h at room temperature. Coverslips were mounted with Prolong Gold Antifade mounting medium with DAPI (cat.#: P36935; Invitrogen). Staining were visualized using a laser-scanning confocal microscope (ZEISS LSM880) with a 40× (NA 1.1) water-immersion objective. Images were analyzed using the open source Fiji software (NIH, Bethesda, U.S.A.).

#### Live-cell imaging of intracellular TRPM7 movement

HEK-293 expressing yellow fluorescent protein (YFP)-tagged WT mouse TRPM7 (WT mTRPM7-YFP) [41]. The transient transfected HEK-293 cells were maintained in DMEM containing 10% FBS. After EGF (50 ng/ml) treatment, fluorescent signal of TRPM7 in HEK-293 cells were recorded for 15 min on an inverted epifluorescence microscope (Axio Observer Z1 Live-Cell imaging system, ZEISS) at excitatory wavelength of 490 nm and emission of 535 nm. Images were quired and analyzed by Zen Pro software (ZEISS).

#### Proximity ligation assays

VSMCs from WKY were seeded on cover glasses (13 mm, Thickness No.0; VWR) at a density of 5 × 10^4^ cells per well in six-well plates. The following day, cells were stimulated with EGF (50 ng/ml) for 5 min with or without 30 min pre-treatment of inhibitors. Cells were fixed with 4% paraformaldehyde in PBS for 15 min at room temperature, and cell membranes were then stained by wheat germ agglutinin (WGA; cat.#: w6748; Invitrogen) for 25 min, followed by permeabilization using 0.1% Triton X-100 (Sigma–Aldrich) in PBS for 10 min at room temperature. Cells were washed with Buffer A (0.01 M Tris-Base, 0.15 M NaCl, 0.05% Tween-20, pH 7.4) and blocked using Blocking Buffer for 1 h at 37°C, followed incubation with primary antibodies EGFR (cat.#: 2646S; Cell Signaling; 1:100) and TRPM7 (cat.#: MA5-27620; Invitrogen; 1:100) overnight at 4°C. The following day, proximity ligation assay (PLA) was performed according to the manufacturer’s protocol using the Duolink Detection Kit (Sigma–Aldrich). Briefly, cells were washed using Buffer A and incubated with Duolink® In Situ PLA® Probe Anti-Mouse PLUS (cat.#: DUO92001) and Duolink® In Situ PLA® Probe Anti-Rabbit MINUS (cat.#:DUO92005) at 37°C for 1 h to label TRPM7 and EGFR, respectively. Circular DNA molecules were formed in Ligation Buffer (working dilution 1:5) containing DNA Ligase (1:40) at 37°C for 30 min, and were amplified in Amplification Buffer (1:5) containing the rolling circle polymerase (1:80) at 37°C for 100 min. Coverslips were mounted with Duolink® In Situ Mounting Medium with DAPI (cat.#: DUO82040; Sigma–Aldrich). Images were acquired using a laser-scanning confocal microscope (LSM 5 PASCAL ZEISS) under an oil immersion objective (63×/1.4 NA). In order to detect all PLA signals, a series of Z-stack images were collected and were analyzed by ImageJ software.

### Statistical analysis

Results were expressed as mean ± standard error of the mean (SEM). Statistical analysis was conducted using GraphPad Pad 5.0 (GraphPad Software Inc, San Diego, CA). Unpaired and two-tailed *t* test or one-way analysis of variance (ANOVA) with Dunnett’s or Tukey’s post-test were used as appropriate. Results of statistical tests with *P*-value less than 0.05 were considered significant.

## Results

### EGF regulates TRPM7 but not TRPM6 and SLC41A1 through EGFR and c-Src dependent mechanisms in rVSMCs

**Figure 1:**
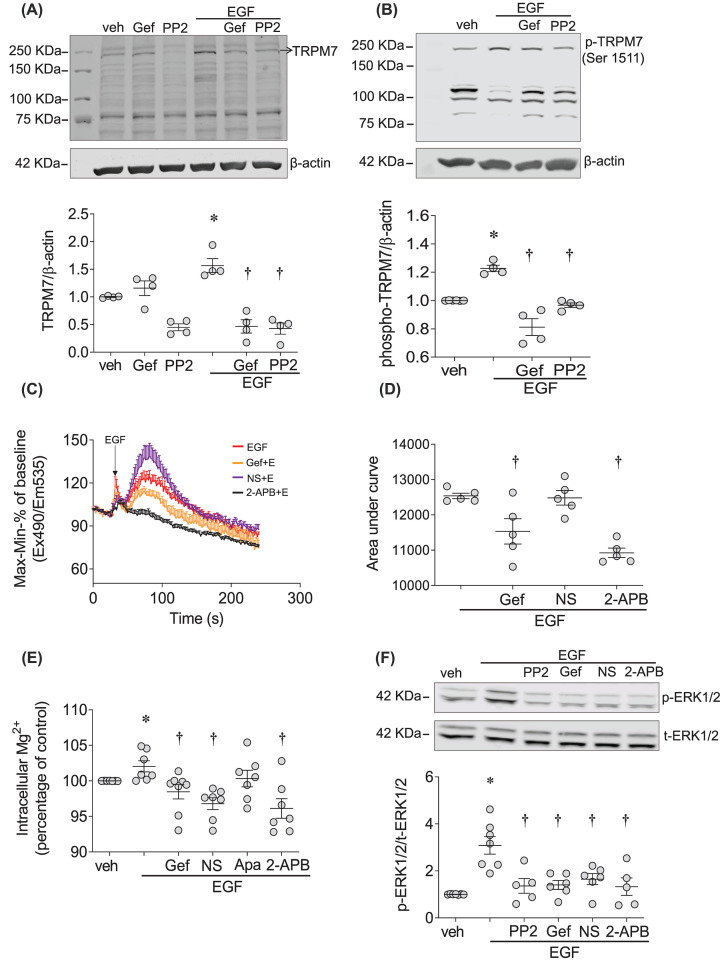
EGF regulates TRPM7 and signaling through EGFR and c-Src in rVSMCs (**A**,**B**) rVSMCs were treated with EGF (50 ng/ml) in the presence and absence of EGFR inhibitor gefitinib (Gef, 1 µM) and c-Src inhibitor PP2 (10 µM). (A) TRPM7 expression, normalized to β-actin, after 24 h stimulation and (B) TRPM7 phosphorylation, normalized to β-actin, after 5-min stimulation. Upper panels are representative immunoblots. (**C**,**D**) Intracellular Ca^2+^ levels ([Ca^2+^]_i_) in rVSMCs induced by EGF (50 ng/ml) were measured in the presence and absence of gefitinib (Gef, 1 µM), the TRPM7 inhibitor NS8593 (NS, 40 µM) and the non-selective cation channel inhibitor 2-APB (30 µM) in rVSMCs. (D) [Ca^2+^]_i_ data are expressed as (C) fluorescence Cal-520AM-Ca^2+^ signals and (D) calculated as the area under curve. (**E**) Intracellular free Mg^2+^ ([Mg^2+^]_i_ ) in rVSMCs after EGF (50 ng/ml) treatment (5 min) in the presence and absence of gefitinib (1 µM), NS8593 (40 µM), apamin (Apa, 1 µM) and 2-APB (30 µM) were measured by Flow Cytometry using specific Mg^2+^ indicator Magnesium Green AM. Raw data were normalized to percentage differences based on the baseline value (veh). (**F**) ERK1/2 activity was assessed by phosphorylation levels (p-ERK1/2) normalized by total ERK1/2 (t-ERK1/2) expression. Results are mean ± SEM of four to eight experiments. *P<0.05 compared with vehicle control (veh, PBS) and ^†^*P*<0.05 compared with EGF-treated cells.

rVSMCs express EGFR and TRPM7 (Figure 1, Supplementary Figure S1A,B). EGF stimulation for 24 h increased TRPM7 expression in rVSMCs, an effect that was attenuated in cells pretreated with gefitinib and PP2 (Figure 1A). EGF had no effect on expression of other Mg^2+^ channels and transporters like, TRPM6 and SLC41A1 (Supplementary Figure S1C,D).

To determine whether EGF influences the activity of TRPM7-kinase, we assessed the autophosphorylation site at the Ser^1511^ domain, which is critically involved in TRPM7-kinase activation and signaling [39,42]. As shown in Figure 1B, EGF induced a rapid increase (within 5 min) in phosphorylation of TRPM7. This was significantly blunted by gefitinib and PP2 (Figure 1B).

### EGF influences VSMC Mg^2+^ but not Ca^2+^ transients through TRPM7-dependent pathways in rVSMCs

As TRPM7 is a divalent cation channel important in the regulation of Mg^2+^ and Ca^2+^ in VSMC, we investigated its role in EGF-induced changes in intracellular levels of Ca^2+^ and Mg^2+^. In rVSMCs, EGF stimulation increased Ca^2+^ transients, which was reduced by gefitinib and 2-APB (Figure 1C,D). NS8593, a more selective TRPM7 inhibitor [43], did not affect Ca^2+^ influx induced by EGF. Intracellular Mg^2+^ levels were increased in rVSMCs stimulated with EGF (5 min), an effect that was reduced by gefitinib, 2-APB and NS8593 (Figure 1E). Since NS8593 is also an inhibitor of SK channels [43], we used the selective SK channel inhibitor apamin as a control, which failed to change EGF effects on [Mg^2+^]_i_.

### EGF-induced phosphorylation of ERK1/2 involves c-Src and TRPM7

MAPKs, such as ERK1/2 and c-Src signaling are important downstream targets of EGF. EGF-induced phosphorylation of c-Src in rVSMCs. To evaluate the role of c-Src and TRPM7 in this pathway, ERK1/2 phosphorylation was assessed in EGF-stimulated rVSMCs in the presence of PP2, gefitinib, and TRPM7 inhibitors NS8593 and 2-APB. As shown in Figure 1F, EGF significantly increased phosphorylation of ERK1/2 in rVSMCs. These effects were blunted when EGFR, c-Src and TRPM7 were inhibited.

### EGF enhances interaction between EGFR and TRPM7 in a c-Src-dependent manner

**Figure 2:**
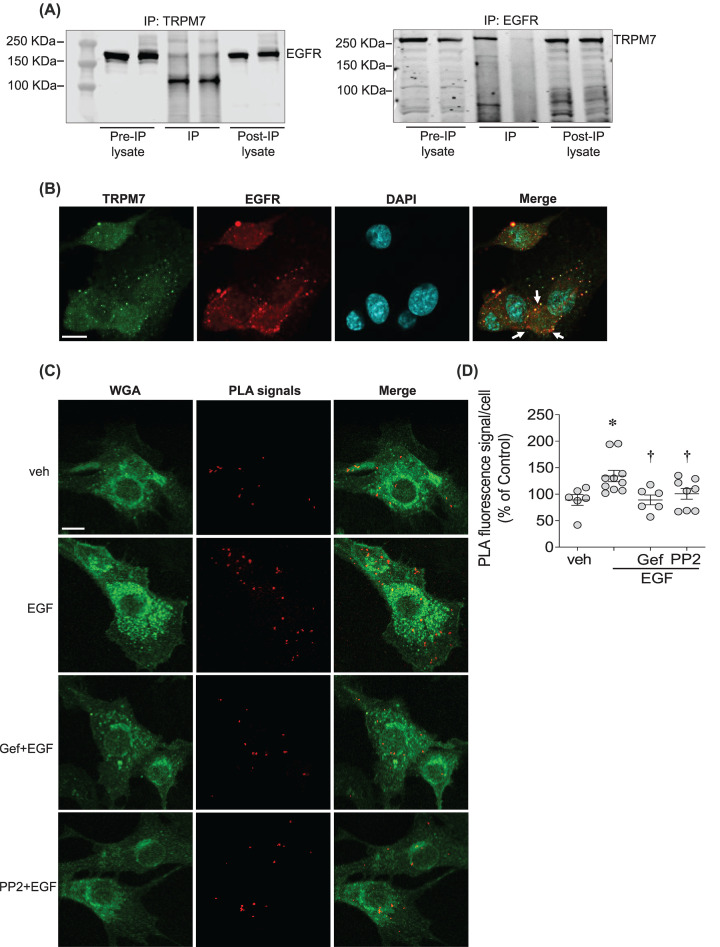
EGF enhances direct interaction between EGFR and TRPM7 in a c-Src-dependent manner (**A**) EGFR was detected when TRPM7 was immunoprecipitated in rVSMCs using anti-TRPM7 antibody (left panel) and TRPM7 was detected when EGFR was immunoprecipitated using anti-EGFR antibody (right panel) (*n*=3–4). (**B**) Representative confocal microscopy images of rVSMCs co-immunostained for TRPM7 (Alexa 488, green) and EGFR (Alexa 555, red). Nuclei were stained with DAPI (*n*=3). (**C**,**D**) PLA was used to visualize and quantify TRPM7-EGFR interaction in rVSMCs stimulated with vehicle (veh), and EGF (50 ng/ml) in the presence and absence of gefitinib (1 µM) and PP2 (10 µM). WGA (Alexa 488, green) was used to stain cell membranes. Red fluorescence is identified as PLA positive signals. Orange fluorescence in the merged figure identifies co-localization. PLA signals were quantified using the Analyse Particles plugin of ImageJ software. For each condition, quantifications were performed from at least 100 cells and expressed as the mean number of signals per cell (percentage of control). Scale bar =10 µm. Arrows indicate co-localization of EGFR and TRPM7. *P<0.05 compared with vehicle control (veh, PBS) and ^†^*P*<0.05 compared with EGF-treated cells.

As shown in Figure 2A, EGFR was detected in rVSMCs when TRPM7 was immunoprecipitated. The association between EGFR and TRPM7 was further confirmed when TRPM7 was detected after EGFR IP. TRPM7–EGFR interaction was also determined by confocal microscopy showing co-localization in the cell membrane (Figure 2B) and by PLA, using specific primary antibodies to EGFR and TRPM7. Figure 2C,D show that in basal conditions, there are PLA positive signals, indicating interaction between EGFR and TRPM7 in the cell membrane. PLA positive signals were increased by EGF stimulation, and reduced by gefitinib and PP2 (Figure 2C,D).

### EGF/EGFR does not influence TRPM7 trafficking in HEK-293 cells

Previous studies in kidney cell lines demonstrated that EGF promotes TRPM6 trafficking from the cytosol to the cell membrane [20]. Having demonstrated that TRPM7 is cell membrane-associated, we questioned whether EGF influences TRPM7 trafficking, as reported for TRPM6 in kidney cells. Using YFP-TRPM7, HEK-293 cells we assessed effects of EGF stimulation on TRPM7 mobilization. As shown in Supplementary Figure S2A,B and Supplementary Video S1 EGF did not influence TRPM7 translocation to the cell membrane, at least for the time period studied (10 min).

### EGF promotes VSMCs migration and proliferation through TRPM7 and ERK1/2

**Figure 3:**
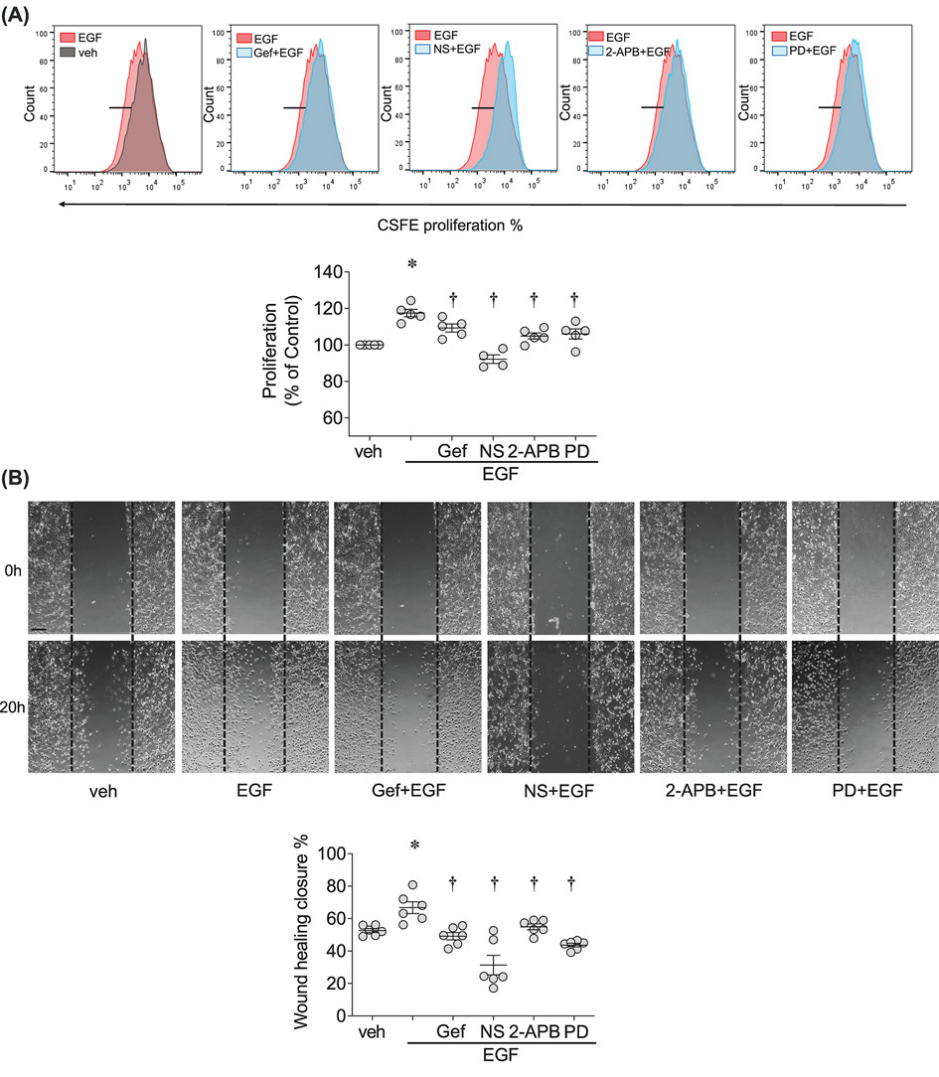
EGF promotes VSMCs migration and proliferation through TRPM7 and ERK1/2 rVSMCs were stimulated with EGF (50 ng/ml), with or without 30 min pre-treatment of gefitinib (Gef, 1 µM), NS8593 (NS, 40 µM), 2-APB (30 µM) and ERK1/2 inhibitor PD98059 (PD, 20 µM). (**A**) Cell proliferation was assessed by CFSE proliferation assay using flow cytometry. Upper panel: Representative flow cytometry histograms. Lower panel: Corresponding scatter-plot graphs showing cell proliferation rate. Raw data were normalized to percentage differences based on the baseline value (veh). (**B**) Cell migration was examined by Scratch-wound assay. Cell migration was calculated as the percentage of wound healing closure at 20 h relative to 0 h time point. Upper panel: Representative images of rVSMCs migration stimulated with EGF in the presence and absence of different inhibitors. Lower panel: Corresponding scatter-plot graphs showing cell migration rate calculated as wound healing closure. Scale bar = 150 µm. *P<0.05 compared with vehicle control (veh, PBS) and ^†^*P*<0.05 compared with EGF. Results represent the mean ± SEM of four to six independent experiments.

Considering that VSMC proliferation and migration are key processes involved in vascular physiology, we assessed the importance of TRPM7 in these functional effects mediated by EGF. Using the CFSE proliferation assay, we found that EGF induced proliferation of rVSMCs in an EGFR–TRPM7–ERK1/2-dependent manner, since EGF stimulated cell growth, effects that were inhibited by gefitinib, TRPM7 inhibitors and the ERK1/2 inhibitor, PD98059 (Figure 3A). To further assess the functional significance of EGF–TRPM7–ERK1/2, we examined VSMC migration using the scratch-wound assay. As shown in Figure 3B, EGF significantly enhanced rVSMCs migration. This was inhibited by gefitinib, NS8593, 2-APB and PD98059.

### EGFR and c-Src are regulated by TRPM7-dependent mechanisms

**Figure 4:**
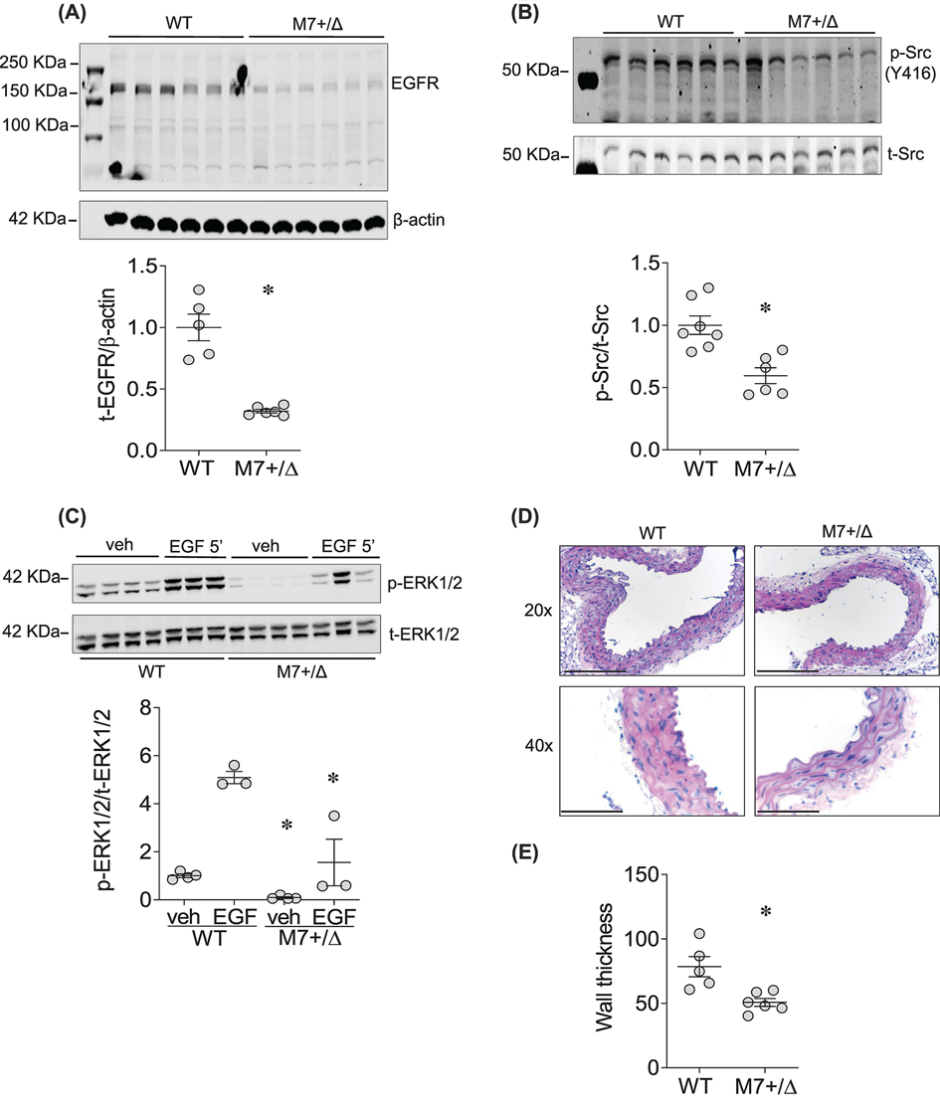
Reduced expression of EGFR is associated with vascular remodeling in TRPM7-deficient mice (**A**) EGFR expression (EGFR) in mVSMCs derived from WT and TRPM7 kinase-deficient mice (TRPM7^+/Δkinase^, M7+/Δ). Protein expression normalized to β-actin. (**B**) c-Src phosphorylation (p-c-Src), normalized by total c-Src expression (t-Src) in mVSMCs derived from WT and TRPM7^+/Δkinase^ mice. (**C**) ERK1/2 phosphorylation in primary cultured mVSMCs derived from WT and TRPM7^+/Δkinase^ (M7+/Δ) mice, at basal levels (veh) and after 5-min treatment with EGF (50 ng/ml). ERK1/2 activity was assessed by phosphorylation level (p-ERK1/2) and normalized by total ERK1/2 expression (t-ERK1/2). Results are mean ± SEM of three to six experiments. (**D**,**E**) Representative images and quantification of aortic wall thickness in WT and TRPM7^+/Δkinase^ mice; 20×: Scale bar =150 µm. 40×: Scale bar =75 µm. Results represent the mean ± SEM of three to seven independent experiments. *P<0.05 compared with WT mice.

Having demonstrated a feedforward loop between EGF/EGFR/c-Src and TRPM7 where interactions between EGFR and TRPM7 may be crucial for EGF signaling, we questioned whether TRPM7 plays a role in regulating vascular EGFR and c-Src. To address this, we evaluated the expression and phosphorylation of EGFR, c-Src and ERK1/2 in VSMCs and intact vascular tissue from mice deficient in TRPM7 (Figure 4A–C). TRPM7^+/Δkinase^ mice, characterized by reduced expression and phosphorylation of TRPM7 (Supplementary Figure S3) had significantly reduced expression and phosphorylation of EGFR and c-Src.

### Reduced phosphorylation of EGFR is associated with vascular remodeling in TRPM7-deficient mice

EGF-induced proliferation and migration of VSMCs are major factors involved in vascular morphology and structure. We investigated the effects of TRPM7 deficiency on EGFR expression and vessel morphology by studying aorta from TRPM7^+/Δkinase^ mice. These processes were associated with significant changes in vascular morphology as evidenced by significantly reduced vascular thickness in TRPM7^+/Δkinase^ mice (Figure 4D,E). Further demonstrating a critical role for EGFR and TRPM7 in vascular homeostasis, we found that expression of PCNA, a pro-prolifeartive marker and Notch3, a cell surface receptor expressed in VSMCs critically involved in vascular structure and development [44], was reduced in mVSMCs and aortas from TRPM7^+/Δkinase^ mice (Supplementary Figure S4A–C).

We also examined aortae from TRPM7^R/R^ mice, another model of TRPM7 deficiency. As shown in Supplemental Figure S5, aortae from TRPM7^R/R^ mice exhibited reduced phosphorylation of EGFR and ERK1/2.

### EGF/EGFR signaling and TRPM7 in hVSMCs

**Figure 5:**
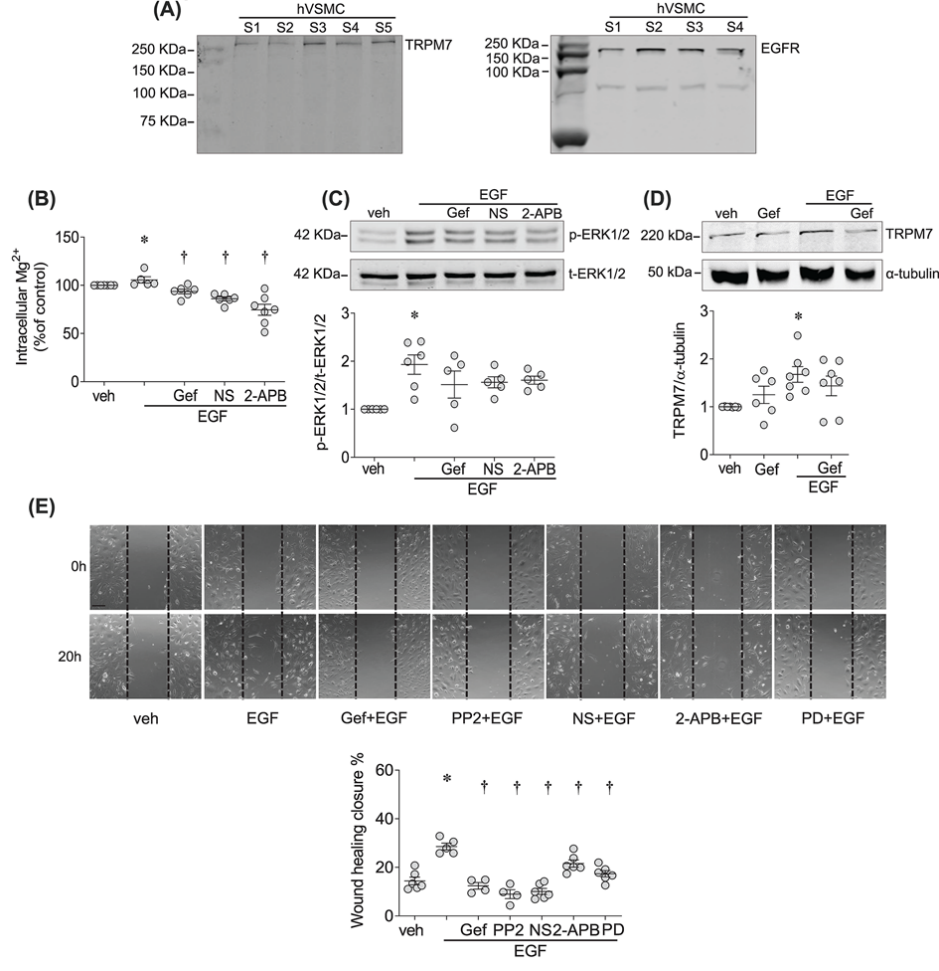
EGF/EGFR signaling and TRPM7 in hVSMCs (**A**) Total cell lysate from hVSMC was probed for TRPM7 (left panel) and EGFR (right panel) expression. Samples S1–S5 were hVSMCs derived from five different donors. (**B**) [Mg^2+^]_i_ in hVSMCs exposed to EGF (50 ng/ml) for 5 min in the absence or presence of gefitinib (Gef, 1 µM), NS8593 (NS, 40 µM) and 2-APB (30 µM). (**C**) ERK1/2 phosphorylation, normalized to total ERK1/2 expression, in hVSMCs exposed to EGF (50 ng/ml) for 5 min in the presence or absence of gefitinib (Gef, 1 µM), NS8593 (NS, 40 µM) and 2-APB (30 µM). (**D**) TRPM7 expression after 5 h treatment with EGF (50 ng/ml) in the presence and absence of gefitinib (Gef, 1 µM). (**E**) Cell migration assessed by Scratch-wound assay in hVSMCs treated with EGF (50 ng/ml) for 20 h in the presence or absence of gefitinib (Gef, 1 µM), PP2 (10 µM), NS8593 (NS, 40 µM), 2-APB (30 µM) and PD98059 (PD, 20 µM). Scale bar = 150 µm. Results represent the mean ± SEM of four to seven independent experiments. *P<0.05 compared with vehicle control (veh, PBS) and ^†^*P*<0.05 compared with EGF.

Next, we investigated whether the novel EGF-TRPM7 pathway observed in cells and tissues from rodents is also evident in humans. We confirmed that primary hVSMCs express TRPM7 and EGFR (Figure 5A). EGF stimulation (5 min) induced a significant increase in [Mg^2+^]_i_ and ERK1/2 phosphorylation, effects that were attenuated by gefitinib, NS8593 and 2-APB (Figure 5B,C). Long-term stimulation (5 h) with EGF increased TRPM7 expression, which was abolished by gefitinib (Figure 5D). To test the functional significance of these events, we assessed EGF-induced migration of hVSMCs. As shown in Figure 5E, EGF stimulation significantly increased migration of hVSMCs. These responses were reduced by gefitinib, PP2, NS8593, 2-APB and PD98059, demonstrating that EGF-induced migration of hVSMC involves c-Src–TRPM7–ERK1/2-dependent pathways.

## Discussion

Our study identifies TRPM7 as a signaling target of EGF in VSMCs. In particular we demonstrate that (i) EGF/EGFR induces phosphorylation of TRPM7, important in VSMC [Mg^2+^]_i_ but not [Ca^2+^]_i_ homeostasis, (ii) EGFR and TRPM7 physically interact on the cell membrane in a c-Src-dependent manner, (iii) EGF/EGFR-mediated activation of TRPM7 involves c-Src and (iv) EGF/EGFR/c-Src signaling promotes VSMC proliferation and migration through TRPM7-regulated ERK1/2-dependent processes. Moreover, we demonstrate that TRPM7 is both downstream and upstream of EGFR, suggesting that EGFR itself is regulated by TRPM7 in a feedforward manner. Together our data define a novel EGF signaling pathway involving c-Src and TRPM7 and show functional interplay between EGFR and TRPM7, important in the regulation of vascular homeostasis.

Vascular effects of EGF are mediated via well-defined signaling pathways involving MAPKs, phosphoinositide-3 kinase (PI3K), PLC-γ, c-Src and transactivation by G protein-coupled receptors [16]. Here we advance the field by delineating a role for TRPM7. This phenomenon seems to be highly regulated and cell-specific, because in VSMCs EGF did not influence TRPM6 or the other Mg^2+^ transporter SLC41A1, but increased activity of TRPM7, whereas in renal tubule epithelial cells, EGFR activation stimulates TRPM6 but not TRPM7 [20].

The molecular significance of TRPM7 as a downstream target of EGF in the vascular system is still unclear but likely relates to its role as a cation channel, since EGF influences cellular Ca^2+^ and Mg^2+^ homeostasis, critically involved in VSMC regulation [8,45,46]. EGF signaling through Ca^2+^, a pivotal second messenger in VSMCs, has been well described [16], but there is a paucity of information about EGF and [Mg^2+^]_i_. The first evidence suggesting a link between EGF and Mg^2+^ homeostasis derived from clinical studies. Individuals with *EGF* mutations were shown to have associated isolated recessive renal hypomagnesemia [29] and cancer patients treated with the EGFR inhibitor cetuximab have reduced serum Mg^2+^ levels that normalize upon therapy withdrawal [25,26]. Our findings in rat and human VSMCs further support the notion that EGF influences intracellular cation homeostasis. This seems to be more important for Mg^2+^ than Ca^2+^ because the TRPM7 inhibitor NS8593 attenuated Mg^2+^ but not Ca^2+^ responses, whereas the non-specific inhibitor of TRP channels 2-APB [47] reduced both Ca^2+^ and Mg^2+^ transients. Corroborating our findings others showed that in cancer cells EGF influences Ca^2+^ in a TRPM7-independent manner probably through other TRP channels [48]. To explore potential molecular mechanisms whereby EGF influences TRPM7, we focused on c-Src, a key downstream kinase of EGFR activation [49,50]. In particular, PP2, a selective Src inhibitor, decreased activity and expression of TRPM7 in VSMC.

To further explore the relationship between EGF signaling and TRPM7, we employed a multidisciplinary approach of co-IP, live-cell imaging, and PLAs in rodent and hVSMCs, vascular tissue from TRPM7-deficient mice and TRPM7-transfected HEK cells. PLA is a powerful technique to detect, localize and quantify protein–protein interaction, which in combination with co-IP and co-localization experiments, clearly indicated EGFR–TRPM7 interaction in VSMCs. Molecular processes regulating this interaction are unclear but may depend on phosphorylation of the respective partner proteins, involvement of adaptor molecules that promote physical association and localization in subcellular compartments. TRPM7 localizes in intracellular vesicles and traffics to the membrane [51]. In our study TRPM7 clearly co-localized with EGFR on the cell membrane in VSMCs. However, in TRPM7-expressing HEK cells TRPM7 was located primarily in intracellular membrane compartments, similar to what has previously been shown for renal TRPM6 [20]. Hence TRPM7 trafficking may be differentially regulated in a cell-specific manner.

The interplay between EGFR and TRPM7 seems to be circuitous because EGFR is both upstream and downstream of TRPM7. This is supported by the findings that gefitinib inhibited phosphorylation of TRPM7, while TRPM7 deficiency was associated with reduced phosphorylation of EGFR and its downstream target c-Src. Of significance, we focused on phosphorylation of c-Src at tyrosine 416 (Y416), which is associated with high kinase activity, and phosphorylation of EGFR at Tyr^845^ (Y845), a c-Src-dependent phosphorylation site [49,50]. Hence EGF/EGFR-induced activation of TRPM7 influences EGFR phosphorylation and may represent a compensatory mechanism to maintain [Mg^2+^] homeostasis and EGF signaling in VSMC, especially in pathological conditions associated with abnormal EGF and/or TRPM7 regulation.

A unique feature of TRPM7 is that is has dual properties functioning as an ion channel and as a cytosolic kinase [1,2,4]. It is therefore not surprising that in addition to regulating Mg^2+^ influx, it participates in downstream signaling and cell function. TRPM7 influences tyrosine kinases, including EGFR and c-Src, as we demonstrate here, as well as MAP kinases [1,4]. Of the MAP kinases, ERK1/2 is especially important in EGF signaling in VSMCs, since it mediates cell growth, migration and contraction [16]. Our study suggests that EGF/EGFR stimulates ERK1/2 signaling through TRPM7-dependent pathways, since pharmacological and genetic inhibition of TRPM7 was associated with reduced EGF-induced ERK1/2 phosphorylation. Functionally, these processes influence VSMC proliferation and migration, since pharmacological inhibition of EGF, c-Src, ERK1/2 and TRPM7 attenuated EGF-stimulated responses. Previous studies demonstrated a role for TRPM7-ERK1/2 in cell proliferation, with variable responses. In endothelial cells, TRPM7 silencing promoted cell growth via ERK1/2, while in mouse cortical astrocytes, TRPM7 knockdown inhibited proliferation through ERK1/2 and c-Jun N-terminal kinases (JNK) [52,53]. To explore the biological significance of our cell-based studies, we examined intact vessels from TRPM7^+/Δkinase^ mice, which have EGFR down-regulation, and showed reduced vascular wall thickness with decreased expression of PCNA and Notch3, markers of VSMC proliferation and differentiation, respectively [44,54]. Our observations of decreased aortic phosphorylation of EGFR and ERK1/2 in TRPM7^R/R^ mice, a second model of TRPM7 deficiency, further validates the notion that EGFR signaling is down-regulated when TRPM7 activity is reduced. Together our findings indicate that EGF/EGFR through c-Src-regulated TRPM7 and ERK1/2 signaling, promotes VSMC proliferation and migration, processes that play a role in vascular remodeling.

**Figure 6:**
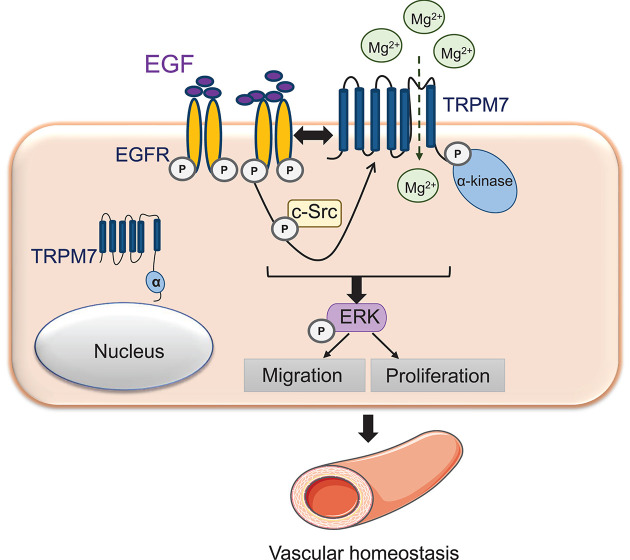
Schematic diagram demonstrating the novel signaling pathway involving EGFR–TRPM7 interaction in VSMCs EGFR activation by EGF results in c-Src activation, which is required for TRPM7 activity (phosphorylation and expression) contributing to Mg^2+^ homeostasis and interaction of EGFR and TRPM7 in the cell membrane. This signaling pathway is required for activation of ERK1/2. Cross-talk between EGF–EGFR and TRPM7 in VSMCs regulates cell migration and proliferation and may play an important role in vascular homeostasis.

In summary, utilizing a combination of pharmacological, biochemical, imaging and molecular biological approaches together with cells and tissues from rodent models we show important cross-talk between EGFR and TRPM7. We provide new evidence that EGFR directly interacts with TRPM7 in VSMCs and that EGF through c-Src regulates TRPM7 in a feedforward system. Functionally these phenomena regulate [Mg^2+^]_i_ homeostasis, ERK1/2 signaling and VSMC function (Figure 6). Of significance our results in rodent VSMCs were recapitulated in hVSMCs, indicating clinical relevance. These findings define a novel pathway in VSMCs and highlight important links between EGF signaling and TRPM7, which may be particularly important in conditions associated with EGFR hyperactivation, such as in cardiovascular disease and cancer.

## Clinical perspectives

- EGF receptor (EGFR) tyrosine kinase signals through multiple pathways and influences cellular cation homeostasis, including Mg^2+^, important in vascular homeostasis and disease. This is clinically relevant because anticancer EGFR tyrosine kinase inhibitors cause hypomagnesemia and hypertension. Processes linking vascular EGF/EGFR and Mg^2+^ are unclear, but the novel Mg^2+^ transporter TRPM7 may be important.
- We demonstrate important interaction between EGFR and TRPM7 in hVSMCs. EGF/EGFR is both upstream and downstream of TRPM7. This interaction involves c-Src and regulates intracellular Mg^2+^ homeostasis, ERK1/2 signaling and VSMC migration and proliferation.
- We identify a novel pathway in VSMCs and highlight important links between EGF signaling and TRPM7. This interplay may be particularly important in conditions associated with EGFR hyperactivation, perturbed cation (Mg^2+^) regulation and vascular dysfunction, such as in hypertension and other cardiovascular diseases.

## Supplementary Material

Supplementary Figures S1-S5

Supplementary Video S1

## Abbreviations

2-APB: 2-aminoethoxydiphenyl borate
CFSE: carboxyfluorescein succinimidyl ester
hVSMC: human vascular smooth muscle cell
mVSMC: mouse vascular smooth muscle cell
PCNA: Proliferating cell nuclear antigen
PLA: Proximity ligation assay
rVSMC: rat vascular smooth muscle cell
