# Supplementary material for: Epidermal growth factor signaling through transient receptor potential melastatin 7 cation channel regulates vascular smooth muscle cell function
Source: Clin Sci (Lond). 2020 Aug 4;134(15):2019–35. doi: 10.1042/CS20200827 (PMC8299307; doi:10.1042/CS20200827)
Supplement: Supplementary Figures S1-S5 [file CS-2020-0827_supp.pdf]

Supplementary Figure S1

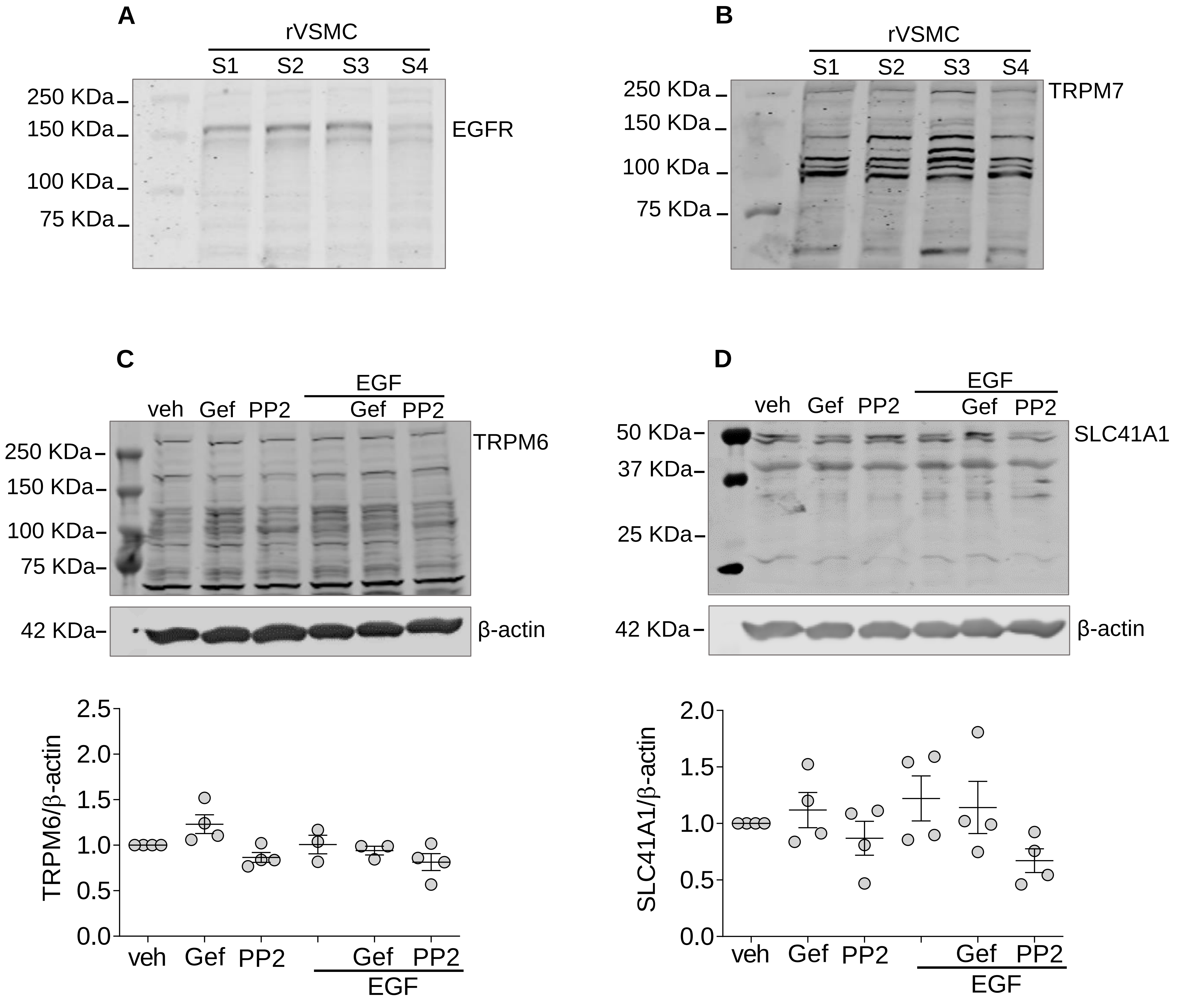

Figure S1. Expression of EGFR and Mg^{2+} transporters in EGF-stimulated rVSMCs. (A, B) Representative immunoblots demonstrating expression of EGFR and TRPM7 in rVSMCs. Sample S1-S4 are rVSMCs derived from 4 independent WKY rats. **(C-E)** rVSMCs were treated with EGF (50 ng/ml, 24 h) in the presence and absence of gefitinib (Gef, 1 µM) and PP2 (10 µM) and protein expression was assessed by immunoblotting. (**C**) TRPM6, and (**D**) SLC41A1. Results represent the mean±SEM of 4 to 6 independent experiments.

Supplemental Figure S2

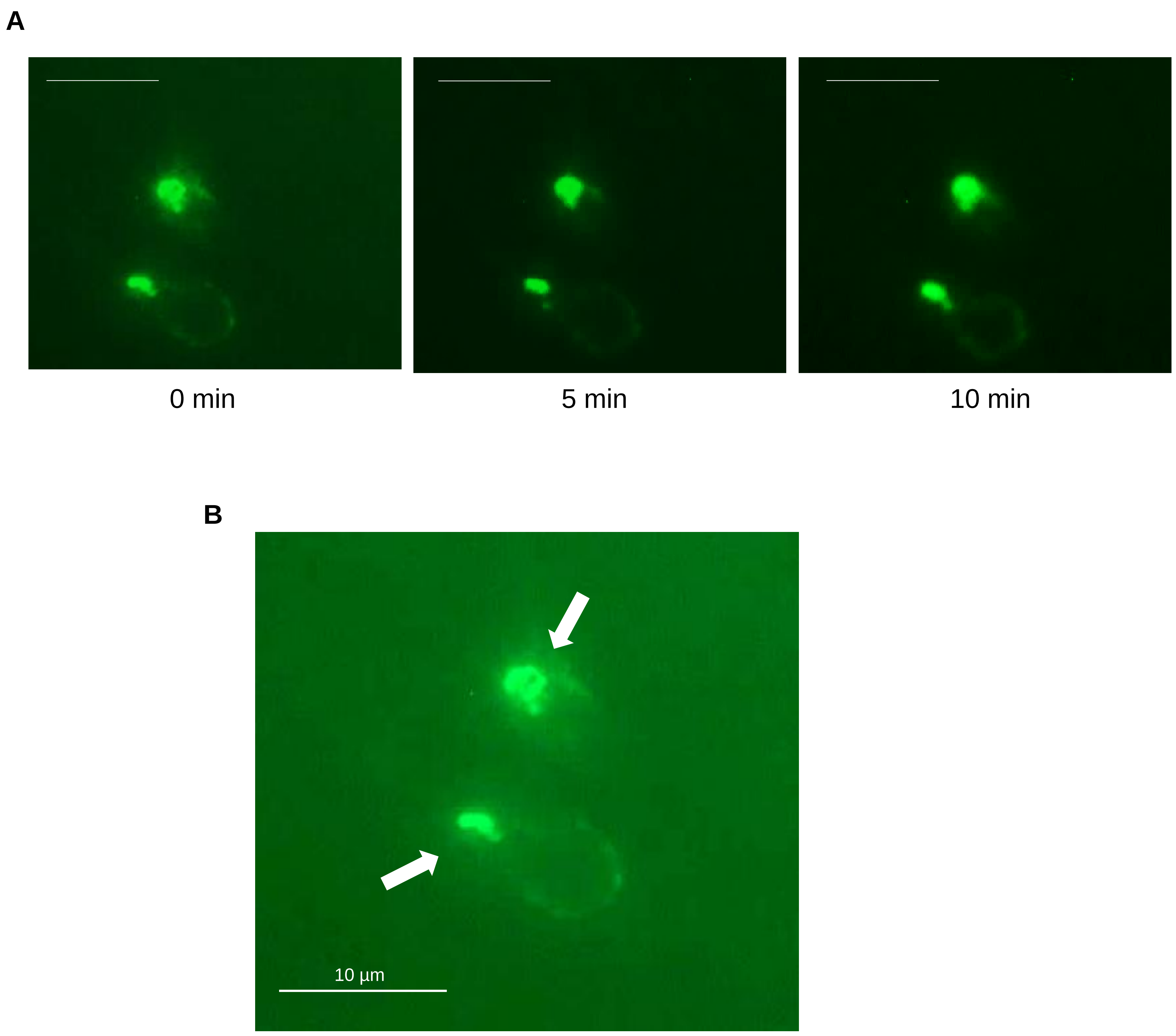

Figure S2. EGF/EGFR does not influence TRPM7 trafficking in HEK-293 cells. HEK-293 cells overexpressing mTRPM7-YFP were visualized by an inverted epifluorescence microscope (Axio Observer Z1 Live-Cell imaging system, ZEISS). TRPM7 movements were recorded after cells were treated with EGF (50 ng/ml) for 10 min. (**A)** shows TRPM7 intracellular location at different time point (0 min, 5min and 10 min) after EGF treatment. (**B)** Shows intracellular TRPM7 movement after EGF stimulation. Images were acquired using 40 x magnification length. Scale bar = 10 µm. Representative images were from 2 independent experiments.

Supplemental Figure S3

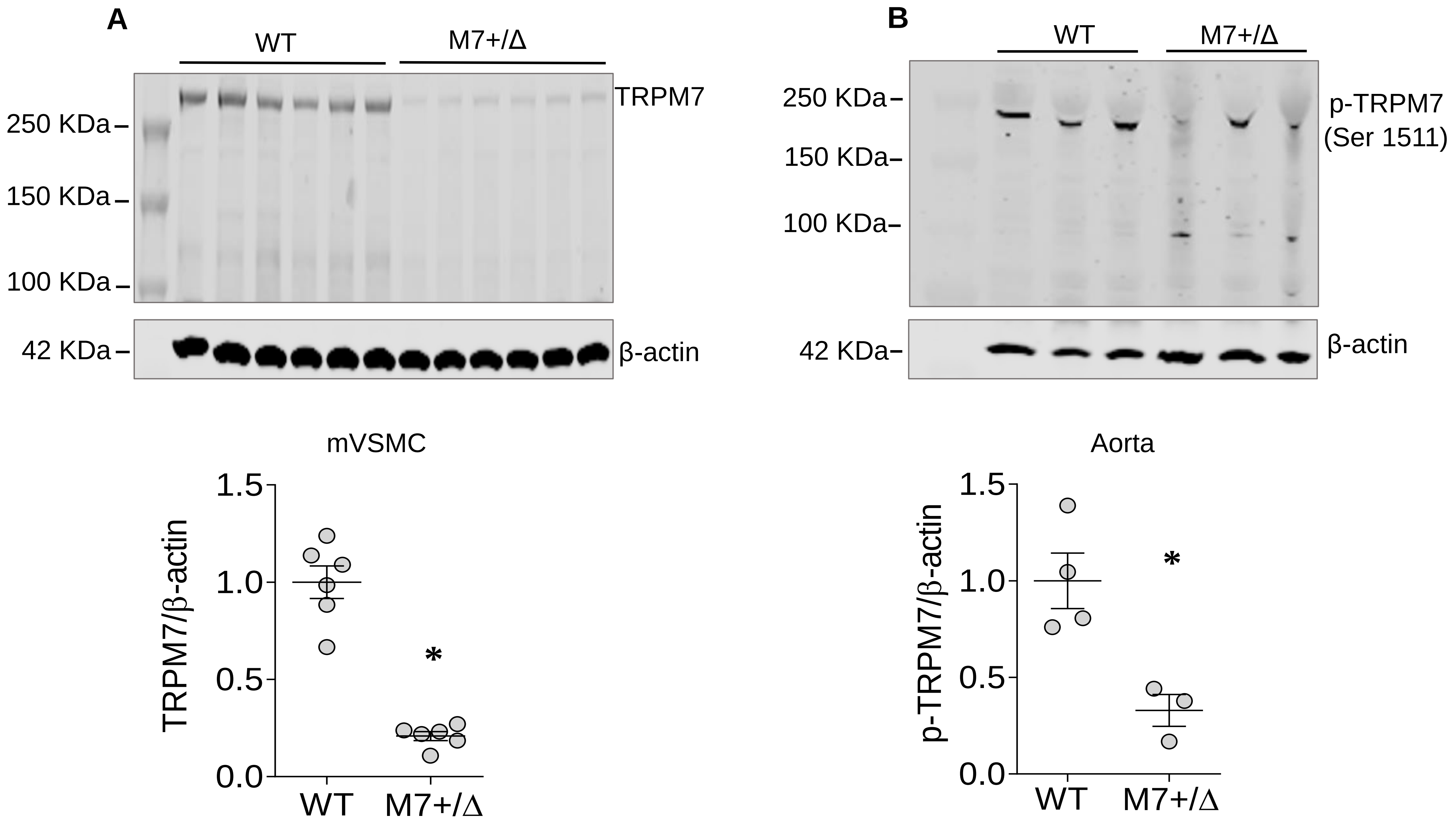

Figure S3. TRPM7$^{+/\Delta kinase}$ mice display reduced expression and phosphorylation of TRPM7. (A) TRPM7 expression in mVSMCs derived from WT and TRPM7$^{+/\Delta kinase}$ mice (M7+/Δ) and (**B**) TRPM7 phosphorylation at serine 1511 residue (Ser 1511) in aortic tissues from WT and TRPM7$^{+/\Delta kinase}$ mice were examined by immunoblotting. Results represent the mean±SEM of 3 to 6 independent experiments. * *P*<0.05 compared to WT.

Supplemental Figure S4

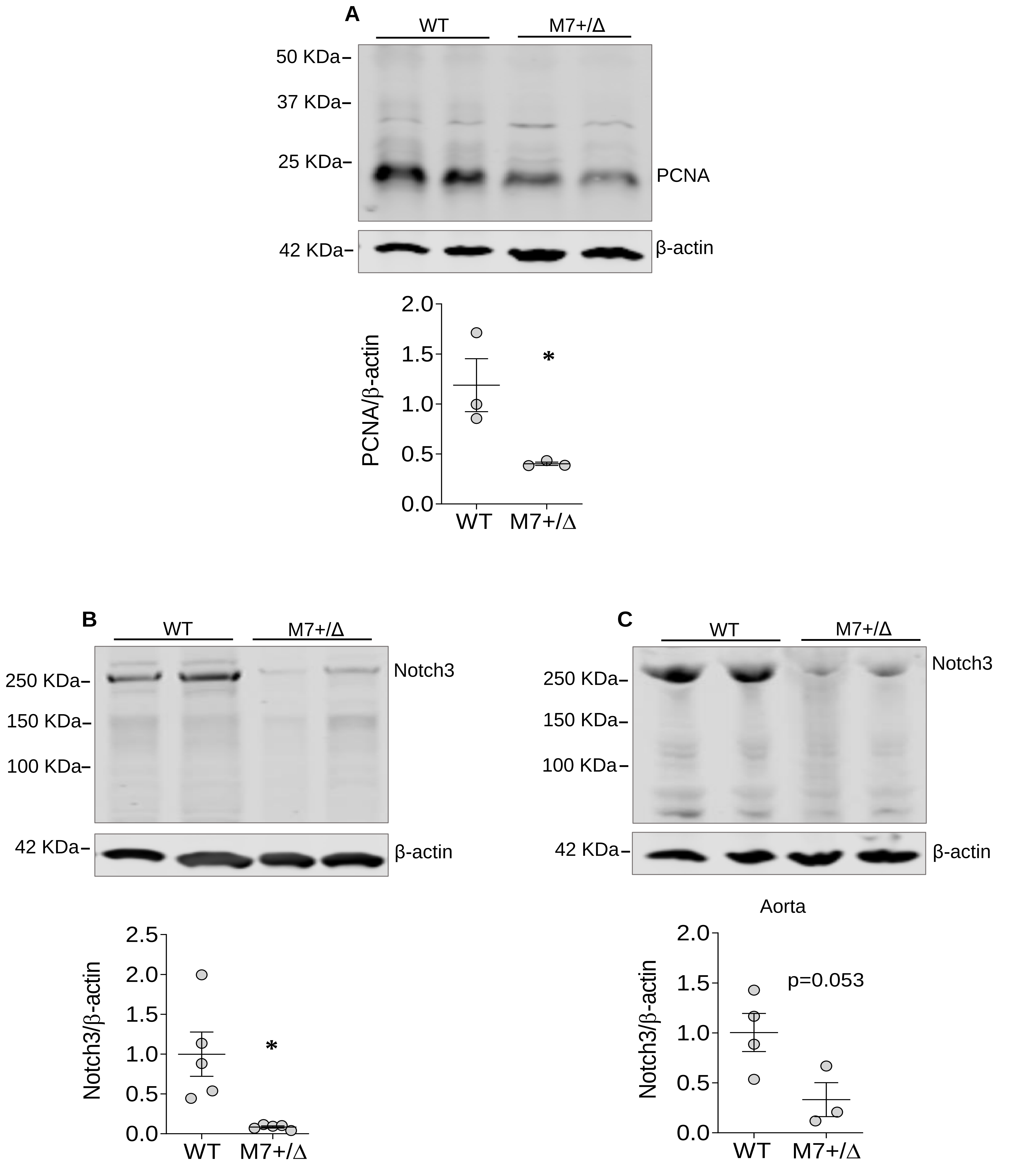

Figure S4. Expression of PCNA and Notch3 is reduced in the vasculature of TRPM7$^{+/\Delta kinase}$ mice. A) PCNA expression in aortic tissues from WT and TRPM7$^{+/\Delta kinase}$ mice (M7+/Δ). **(B)** Expression of Notch3, a VSMC-specific Notch isoform that regulates differentiation, survival and growth, in mVSMCs and (**C**) aortic tissues derived from WT and TRPM7$^{+/\Delta kinase}$ mice assessed by immunoblotting. Protein of interest expressed relative to β-actin. Results represent the mean±SEM of 3 to 5 independent experiments. * *P*<0.05 compared to WT.

Supplemental Figure S5

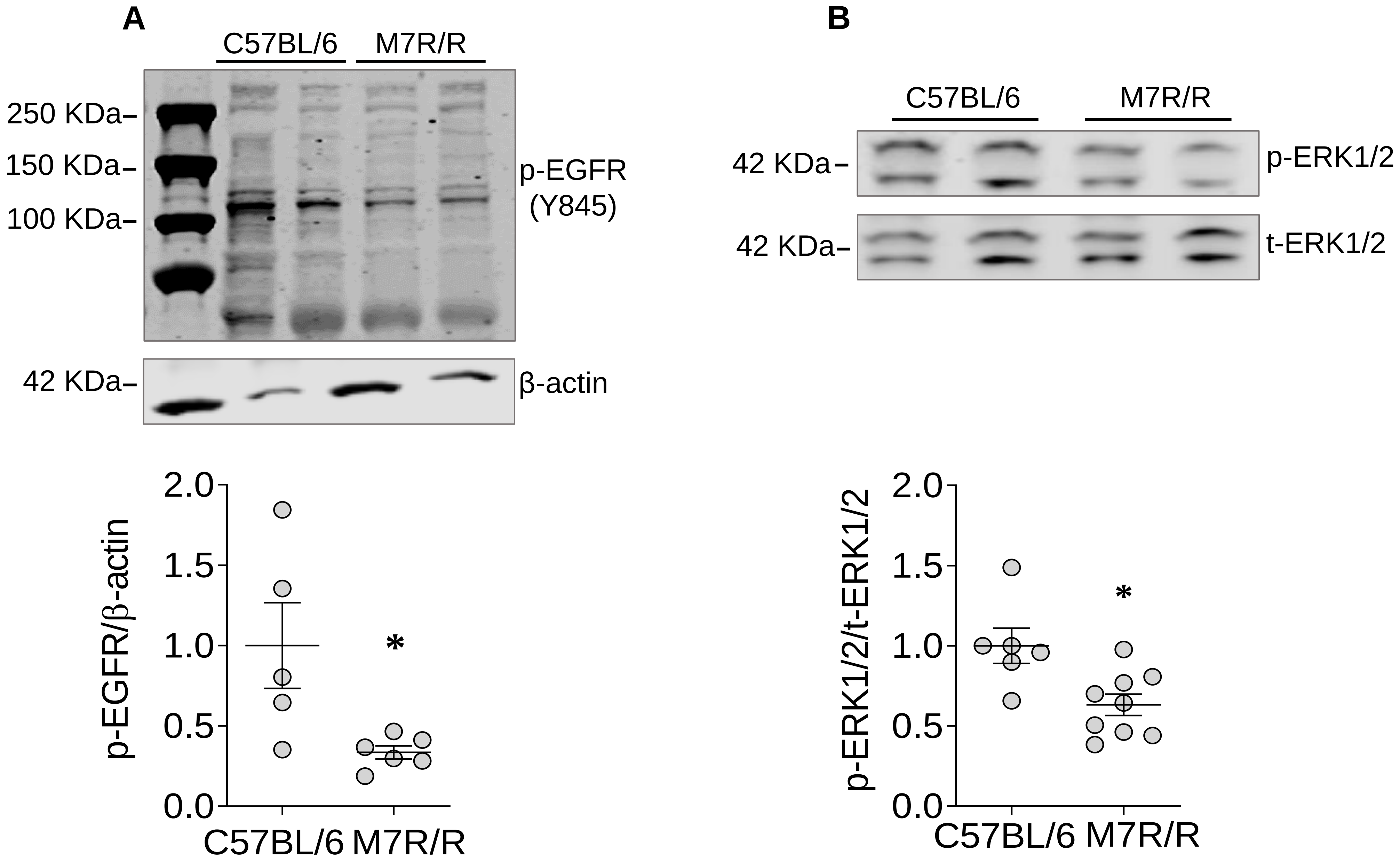

Figure S5. Phosphorylation of EGFR and ERK1/2 in TRPM7-kinase dead mice. (A) Phosphorylation of EGFR at tyrosine 845 (Y845) and **(B)** Phosphorylation of ERK1/2 in aortic tissues from C57BL/6 control mice and TRPM7-kinase dead ($M7^{R/R}$) mice was examined by immunoblotting. Results represent the mean±SEM of 5 to 9 independent experiments. *P<0.05 compared to C57BL/6 mice.
